# Isolated Agenesis of Septum Pellucidum and Adult-Onset Seizure Tendency With Eye Closure Sensitivity

**DOI:** 10.7759/cureus.15463

**Published:** 2021-06-05

**Authors:** Riwaj Bhagat, Elizabeth Smith, Kyle Rizenbergs, Vishwanath Sagi

**Affiliations:** 1 Department of Neurology, University of Louisville School of Medicine, Louisville, USA

**Keywords:** absent septum pellucidum, eye closure sensitivity, new-onset seizure, septum pellucidum, septal agenesis

## Abstract

Septum pellucidum is a thin midline membrane that separates the anterior horns of the lateral ventricle. Agenesis of septum pellucidum (ASP) is considered a continuum of forebrain maldevelopment. Isolated ASP is a rare radiographic finding of unclear significance. We report a case of a 42-year-old male with ASP who presented with a new-onset seizure and eye closure sensitivity seen in the electroencephalogram. Magnetic resonance imaging of the brain confirmed the ASP. In the absence of data about the association between seizure and ASP, further studies are needed to determine its significance.

## Introduction

Septum pellucidum is a midline telencephalic triangular thin membrane separating anterior horns of the right and left lateral ventricles. It is made up of glial cells, neurons, fiber bundles, and veins. Developmentally it forms from the lamina terminalis from 12th to 17th weeks of gestation during the process of corpus callosum and forebrain formation [1]. Agenesis of septum pellucidum (ASP) is regarded as a spectrum of maldevelopment of forebrain structures, which is frequently associated with septo-optic dysplasia (SOD) that consists of a triad of midline brain defects, optic nerve hypoplasia, and hypopituitarism [1,2]. We report an adult patient with isolated ASP that presented with new-onset seizure accompanied by abnormal electro-encephalogram (EEG) findings.

## Case presentation

A 42-year-old male was brought into the hospital by his wife following an episode of confusion, which started four hours prior to arrival. Upon bending down at work, he experienced brief self-limited lightheadedness and blurry vision. Shortly after, he lost consciousness, fell back on the couch and remained unconscious for less than a minute. The patient felt well enough to drive himself home after the episode, but he could not remember the way back to his house and took nearly two hours to navigate home. His wife found him parked in the middle of the street with his car door open and word-finding difficulty at that time. The patient’s confusion resolved en-route to the hospital.

His medical history included hypertension, hyperlipidemia, diabetes mellitus, and cerebral palsy. He had no personal or family history of epilepsy, stroke or neurological disorders. He denied consumption of tobacco, alcohol, or illicit drugs. His vital signs were normal with negative orthostatic. He was 5 foot 7 inches tall, with no craniofacial abnormalities. The remainder of the general physical examination was normal. Neurological examination showed minimal left-hand dysmetria, otherwise higher mental status, language, cranial nerves, motor, sensory, deep tendon reflexes, and gait were normal.

**Figure 1:**
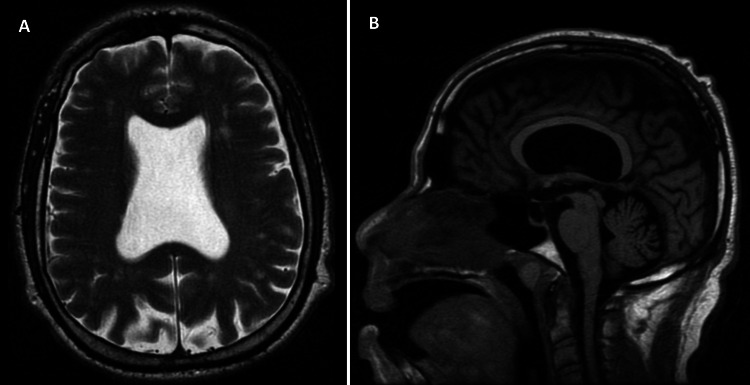
Magnetic resonance imaging of brain T2-weighted axial section (A) and T1- weighted sagittal section (B) show the absence of septum pellucidum in the lateral ventricle.

Initial blood workup showed a glucose level of 188 mg/dL (Ref: 70-110 mg/dL), normal complete blood count, and a comprehensive metabolic panel including electrolytes, liver function, and renal function test. His urine toxicology and thyroid function test were unremarkable. Computed tomography (CT) of the head showed absent septum pellucidum and prominent lateral ventricles. CT angiography of the head and neck was unremarkable. Magnetic resonance imaging of the brain showed the absence of septum pellucidum, intact optic nerve, and pituitary gland (Figures 1A, 1B).

**Figure 2:**
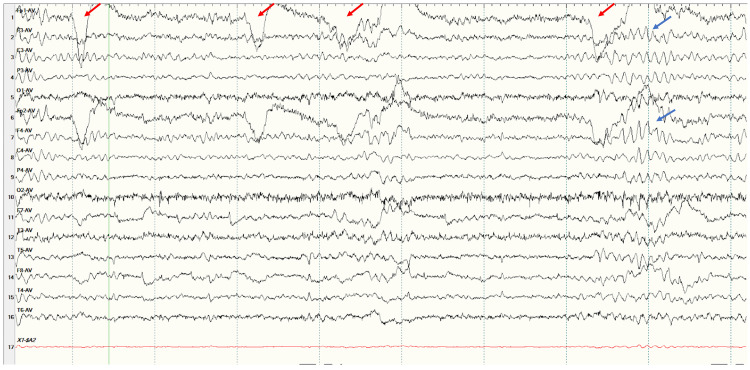
Electroencephalogram with the longitudinal bipolar montage in average reference recording showing rhythmic 6-8 Hz sharp discharges (blue arrow) with bilateral frontal central predominance following eye blinks (red arrow).

Transthoracic echocardiogram and electrocardiogram were unremarkable. EEG was obtained that showed recurrent bursts of high-voltage rhythmic sharply contoured alpha frequency discharges with bilateral central predominance lasting one to two seconds seen mostly after eye closure suggesting eye closure sensitivity (ECS) with the tendency for generalized seizure (Figure 2).

The patient was started on levetiracetam 750 milligrams twice daily. On a yearly follow-up, the patient reported being compliant on his medication and seizure-free.

## Discussion

ASP is not typically seen as an isolated finding. Borkowski‐Tillman et al. found that ASP can occur isolated or in combination with other developmental abnormalities such as corpus callosum agenesis, schizencephaly, holoprosencephaly, and ventriculomegaly [2]. Isolated ASP is associated with a favorable outcome but that ASP associated with other central nervous system findings can predispose to a high risk of abnormal neurodevelopment [2]. Secondary, or acquired, causes of ASP are frequently due to long-standing hydrocephalus secondary to leptomeningitis, trauma, and porencephaly [1]. However, different authors suggest that isolated ASP is extremely rare and that some kind of subtle cortical migration disorder, or SOD, may be present but not visible on brain MRI [3].

Septum pellucidum has an intimate association with structures of the limbic system such as the amygdala, fornix, hippocampus, hypothalamus, and mammillary bodies. The bundled fibers that are contained within its structure serve as highways for the transduction of information between the hypothalamus, hippocampus, and reticular formation suggesting that ASP may play a role in homeostasis and self-regulation [1,4]. However, the precise functional capacity of the septum pellucidum has not been firmly established nor has its role in the pathogenesis of disease states.

Eye closure-induced transient epileptiform changes in the EEG are known as ECS. It has been associated with multiple epilepsy syndromes. It is independent of photosensitivity. Patients with ESC have a good prognosis after initiation of anti-epileptic therapy [5].

Our case with isolated ASP had a dyscognitive episode and ECS detected in EEG, suggesting likely hood of seizure disorder. His seizure was responsive to anti-epileptic therapy and was seizure-free for a year on levetiracetam. No conclusive literature exists on the role of ASP in the pathogenesis of seizures. There are reports that ASP in and of itself is not harmful. It is only when the malformations extend into surrounding structures, such as the fornix, that may involve neurological complications [6] . Neuropsychiatric disorders such as schizophrenia, psychosis, and bipolar disorder have also been linked to ASP [7,8]. Further studies are required to better ascertain the role of ASP in chronic neurological disorders.

## Conclusions

Our case emphasizes the possible association of ASP and seizures supported by an epileptiform EEG pattern. However, a single case can serve only as an “existence proof,” not as an indication of what is usual. Further research is needed regarding this association.
